# Anatomical Network Analysis Shows Decoupling of Modular Lability and Complexity in the Evolution of the Primate Skull

**DOI:** 10.1371/journal.pone.0127653

**Published:** 2015-05-19

**Authors:** Borja Esteve-Altava, Julia C. Boughner, Rui Diogo, Brian A. Villmoare, Diego Rasskin-Gutman

**Affiliations:** 1 Theoretical Biology Research Group, Cavanilles Institute of Biodiversity and Evolutionary Biology, University of Valencia, 46071, Valencia, Spain; 2 Department of Anatomy and Cell Biology, University of Saskatchewan, Saskatoon, SK, Canada; 3 Department of Anatomy, Howard University College of Medicine, Washington, DC, United States of America; 4 Department of Anthropology, University of Nevada Las Vegas, Las Vegas, NV, United States of America; 5 Department of Anthropology, University College London, London, United Kingdom; Santa Fe Institute, SPAIN

## Abstract

Modularity and complexity go hand in hand in the evolution of the skull of primates. Because analyses of these two parameters often use different approaches, we do not know yet how modularity evolves within, or as a consequence of, an also-evolving complex organization. Here we use a novel network theory-based approach (Anatomical Network Analysis) to assess how the organization of skull bones constrains the co-evolution of modularity and complexity among primates. We used the pattern of bone contacts modeled as networks to identify connectivity modules and quantify morphological complexity. We analyzed whether modularity and complexity evolved coordinately in the skull of primates. Specifically, we tested Herbert Simon’s general theory of *near-decomposability*, which states that modularity promotes the evolution of complexity. We found that the skulls of extant primates divide into one conserved cranial module and up to three labile facial modules, whose composition varies among primates. Despite changes in modularity, statistical analyses reject a positive feedback between modularity and complexity. Our results suggest a decoupling of complexity and modularity that translates to varying levels of constraint on the morphological evolvability of the primate skull. This study has methodological and conceptual implications for grasping the constraints that underlie the developmental and functional integration of the skull of humans and other primates.

## Introduction

Modularity and complexity are two biological phenomena that occur together in the development and evolution of the skull in humans as well as in non-human primates [1]; however, most studies tend to overlook the tight relationship between these two phenotypic features [2]. On the one hand, the study of phenotypic modularity is most commonly directed toward validating genetic, developmental, and functional hypotheses of morphological integration, through the analysis of shape covariation using geometric morphometrics [3]. For example, this approach has been used in many cases to test for the division of the skull of primates in three modules: face, vault, and basicranium [4–6]. On the other hand, classic studies on phenotypic complexity have focused on the statistical description of the number of cell types [7] or the number of anatomical parts [8–10]. Using this latter approach in comparative studies of the skull has led researchers to interpret the losses and fusions of bones during vertebrate evolution (Williston’s law) as processes leading to a loss in the complexity of the skull [11–13]. Accordingly, one might argue that the primate skull has become simpler due to the fusion of the frontal bones (from the last common ancestor of Primates to that of Haplorrhini), and the loss of premaxillary bones (from the last common ancestor of Hominoidea to *Homo*).

The lack of a common conceptual and methodological framework to assess modularity and complexity in morphology has prevented a better understanding of how phenotypic modules evolve within, or as a consequence of, an also-evolving complex organization of the skull. Herbert Simon’s *near-decomposability hypothesis* suggested ways to tackle this relationship between modularity and complexity in evolution [14,15]. In Simon’s view, modularity promotes the evolution of complexity through tinkering and robustness, due to the quasi-independence (near-decomposability) of each module, as a consequence of a hierarchical arrangement of parts, granting systems greater evolvability. Many authors have recognized modularity as the most important feature of morphological organization, facilitating the evolution and emergence of complex phenotypes [16–18]. Simon [14] described this concept in theoretical terms applying his well-known watchmaker metaphor, where a modularly designed watch would be able to accept a much greater amount of perturbation during assembly. Linking with Simon’s ideas, West-Eberhard ([19], pg. 182) pointed out that the hierarchical organization of biological development also contributes to evolvability by generating relatively independent functional or structural units that can vary without interfering with other such units [20]. Our definition of morphological evolvability here matches that of Brigandt [21] as an EvoDevo concept that “refers to the capacity of organisms to generate heritable phenotypic variation” fostered by the quasi-independence among parts.

**Fig 1:**
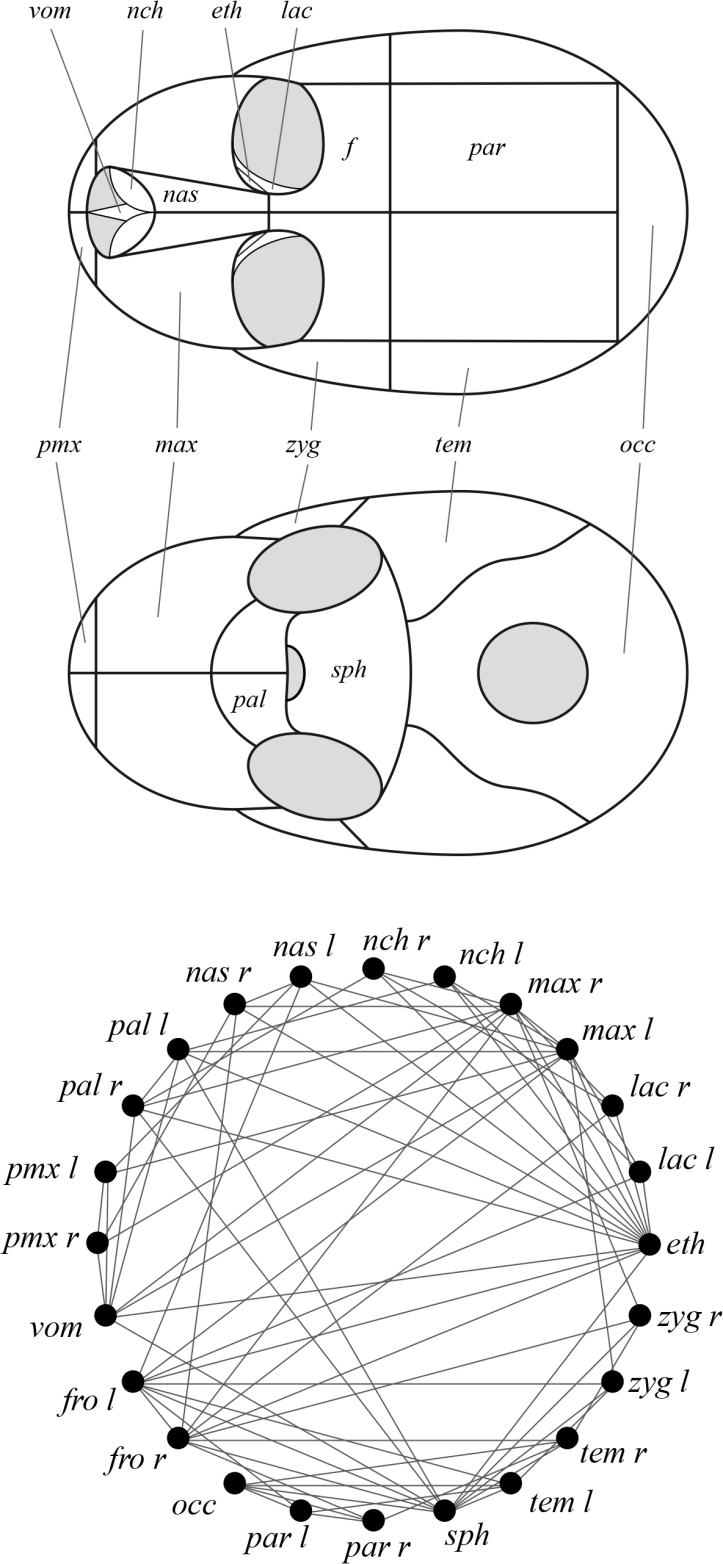
Schema of the skull of primates formalized as a network. An anatomical network represents the bones and physical joints (i.e. sutures and synchondroses) of the skulls as nodes and links in a network model. Because these physical joints are primary sites of bone growth and diffusion of stress forces, topological relations also capture developmental and functional co-dependences among bones [27]. The analysis of these anatomical networks helps uncover the morphological organization of the skull regardless of variation in its shape and size. *Labels*: *eth*, *ethmoid; fro*, *frontal; lac*, *lacrimal; max*, *maxilla; nas*, *nasal; nch*, *nasal concha; occ*, *occipital; pal*, *palatine; par*, *parietal; pmx*, *premaxilla; sph*, *sphenoid; tem*, *temporal; vom*, *vomer; zyg*, *zygomatic; l*, *left; r*, *right*.

In a series of recently published articles, we have used Anatomical Network Analysis (AnNA) as a common framework to independently study phenotypic modularity and complexity in the vertebrate skull (reviewed in [22]) and provide new insights on the musculoskeletal organization of the human head [23]. AnNA is the study of the connectivity patterns that define the morphological organization of anatomies using tools and statistics borrowed from network theory. AnNA uses network models to formalize the bones and physical contacts of the skull as the nodes and links of the network (Fig 1). Computational methods are then used to analyze how these network models organize, and to directly address issues pertaining to modularity, complexity, and evolvability in macroevolution. Assessing morphological organization using connectivity patterns is possible because bone contacts act as primary sites of bone growth, remodeling, and diffusion of stress forces [24–26]. In the context of AnNA, heterogeneous patterns of connections among bones (i.e. patterns in which the number of connections for each bone vary in a spectrum of poorly to highly connected, according to some frequency distribution) define modules as groups of bones with more contacts to bones within the module than outside it [27,28], whereas the richness of bone contacts (e.g. its number and the formation of triangular-loop motifs) is used as a proxy of the skull complexity [29,30].

Some previous studies applying AnNA in a phylogenetic context have revealed that the loss and fusion of bones in tetrapods actually increases the overall complexity of the skull [29], rather than simplifying it as proposed by previous studies of other authors (see above). This result is a consequence of defining complexity by richness of interaction among bones (e.g. the number of bone contacts and the formation of triangular-loop motifs), instead of merely by the number of bones. Moreover, Esteve-Altava and co-workers [30] proposed a specific mechanism underlying this increase in complexity: the random loss of bones with fewer contacts to other bones, and the preferred fusion of bones with more contacts to each other. Interestingly, this reduction in bone number was also concomitant with an increase in specialization and disparity of skull bones [30], which Gregory [31] called anisomerism. AnNA quantifies anisomerism as heterogeneity in the number of bone’s connections [29]. Because anisomerism provides the skull with distinct parts, different from each other, it can also be considered as a measure of complexity.

Other previous studies have used AnNA to identify the modular organization of the human skull [27], and to characterize the modularity-complexity interplay that takes place during ontogeny to produce the adult human skull [28]. These studies identified two connectivity modules in the skull of modern humans that behave as units of allometric growth [27]: a facial module with a hierarchical arrangement of bones in smaller sub-modules (or blocks) around the ethmoid bone, and a cranial module with a pseudo-regular arrangement of bones around the sphenoid bone. Moreover, together the ethmoid and the sphenoid bones participate in almost 40% of the total bone-bone contacts of the human skull, and are thus arguably of developmental and functional significance. It is not yet clear whether the same patterns hold true in the skulls of non-human primates; here, we will analyze these patterns to get a better understanding of the evolutionary processes that ultimately led to the derived form of the human skull.

**Fig 2:**
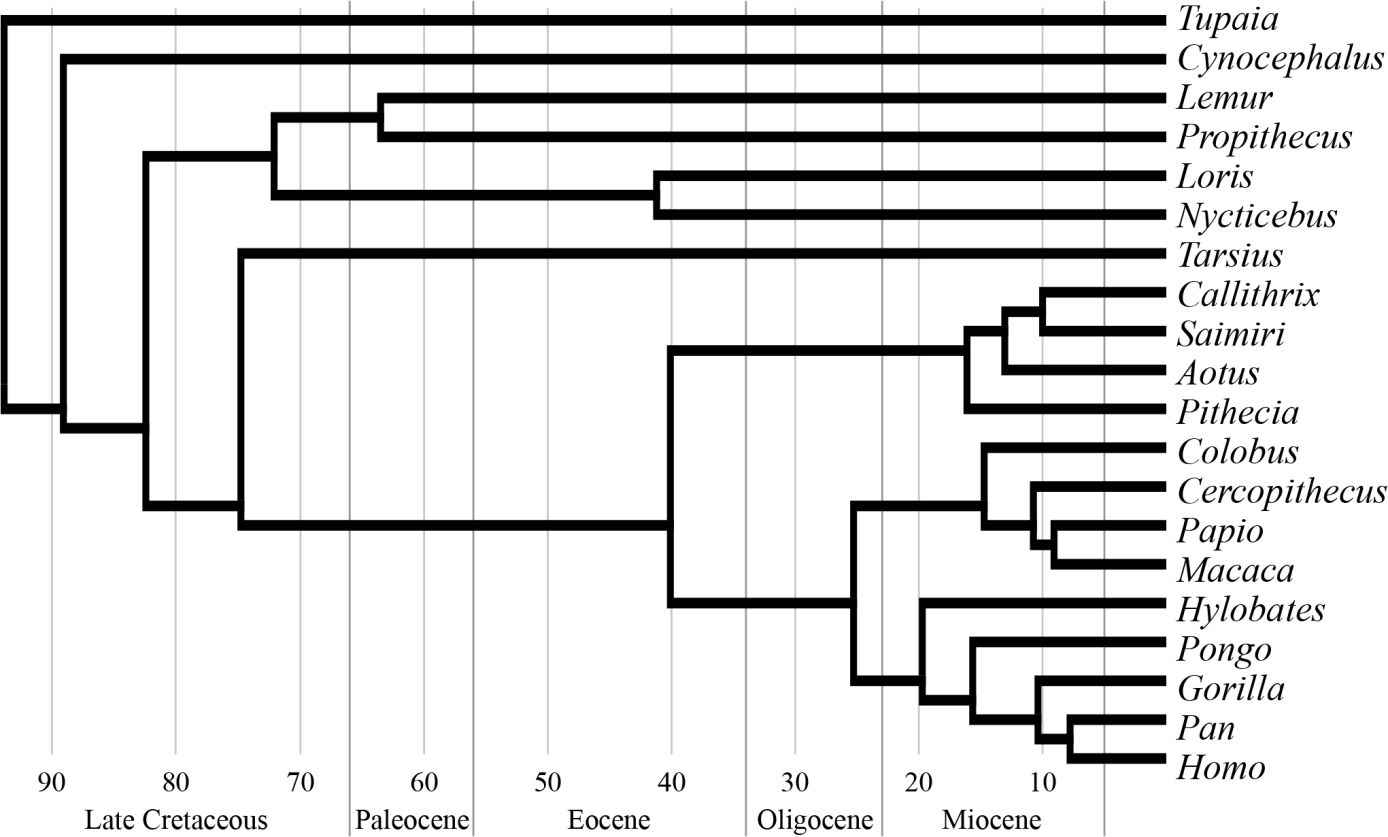
Phylogenetic relations of the 20 taxa studied. Calibration of branch length follows molecular dating [32].

Our present analysis is therefore, to our knowledge, the first unified study of the phenotypic modularity and complexity of the skull for representatives of all major primate taxa, as well as of the two living groups more closely related to Primates: Dermoptera (or colugos represented by *Cynocephalus*) and Scandentia (or tree shrews, represented by *Tupaia*) (Fig 2). We used AnNA to characterize the modular organization of the skull in these taxa, and to test specifically whether modularity correlates positively with complexity (*near-decomposability hypothesis*) in their skull, in an evolutionary context.

## Results

### Phenotypic modules in skull networks

**Fig 3:**
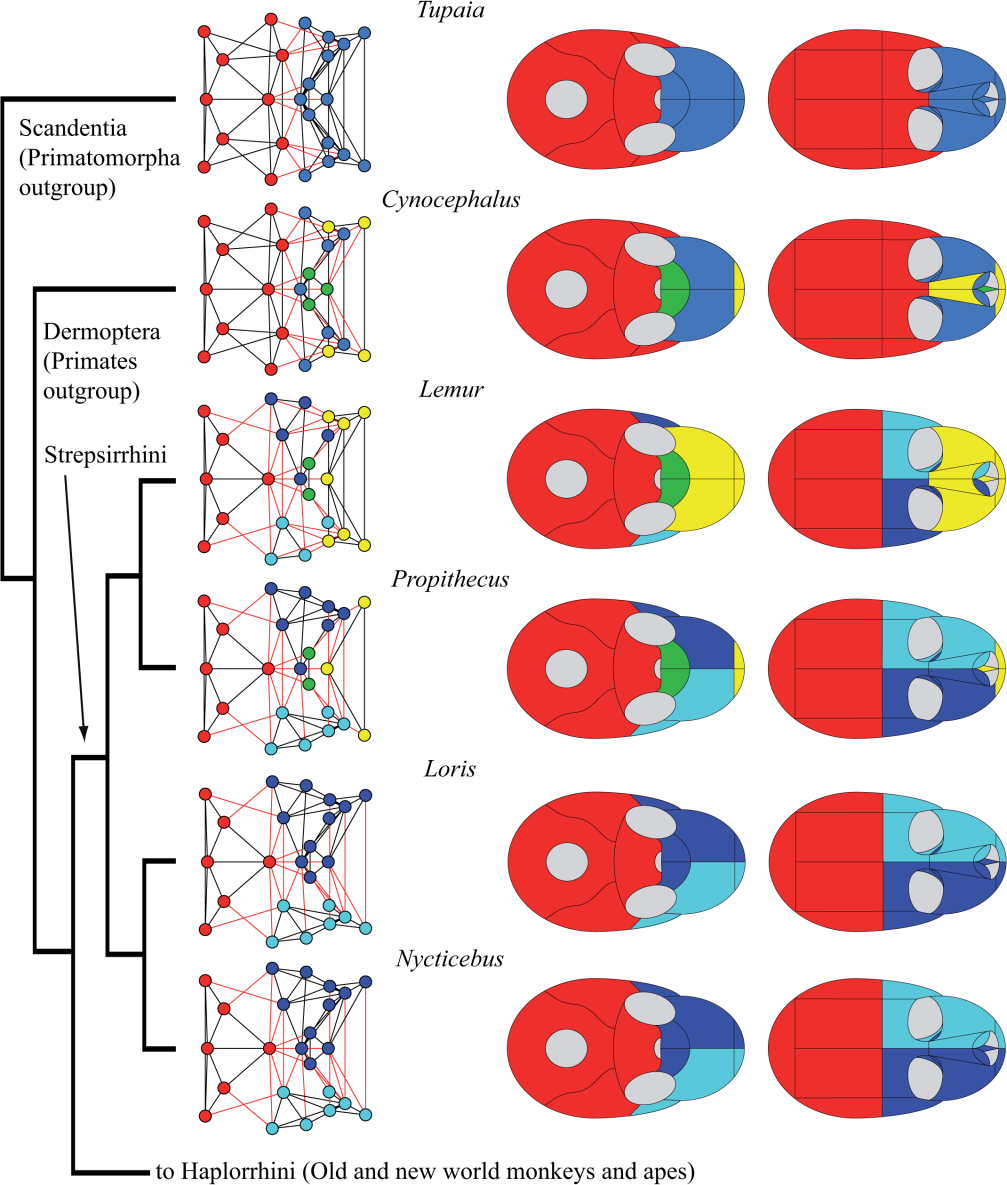
Connectivity modules identified in the skull of outgroup taxa and Strepsirrhini. The four main types of modules: midfacial (in *blue*), palatal (in *green*), premaxillary (in *yellow*) and neurocranial (in *red*) are already present in the skull of *Tupaia* and *Cynocephalus*. The skulls of Strepsirrhini (*left*) show a conserved composition of the cranial module: occipital, sphenoid, parietals, and temporals. The midfacial module is divided into left and right specular modules. Strepsirrhini vary in the formation of palatal and premaxillary modules.

**Fig 4:**
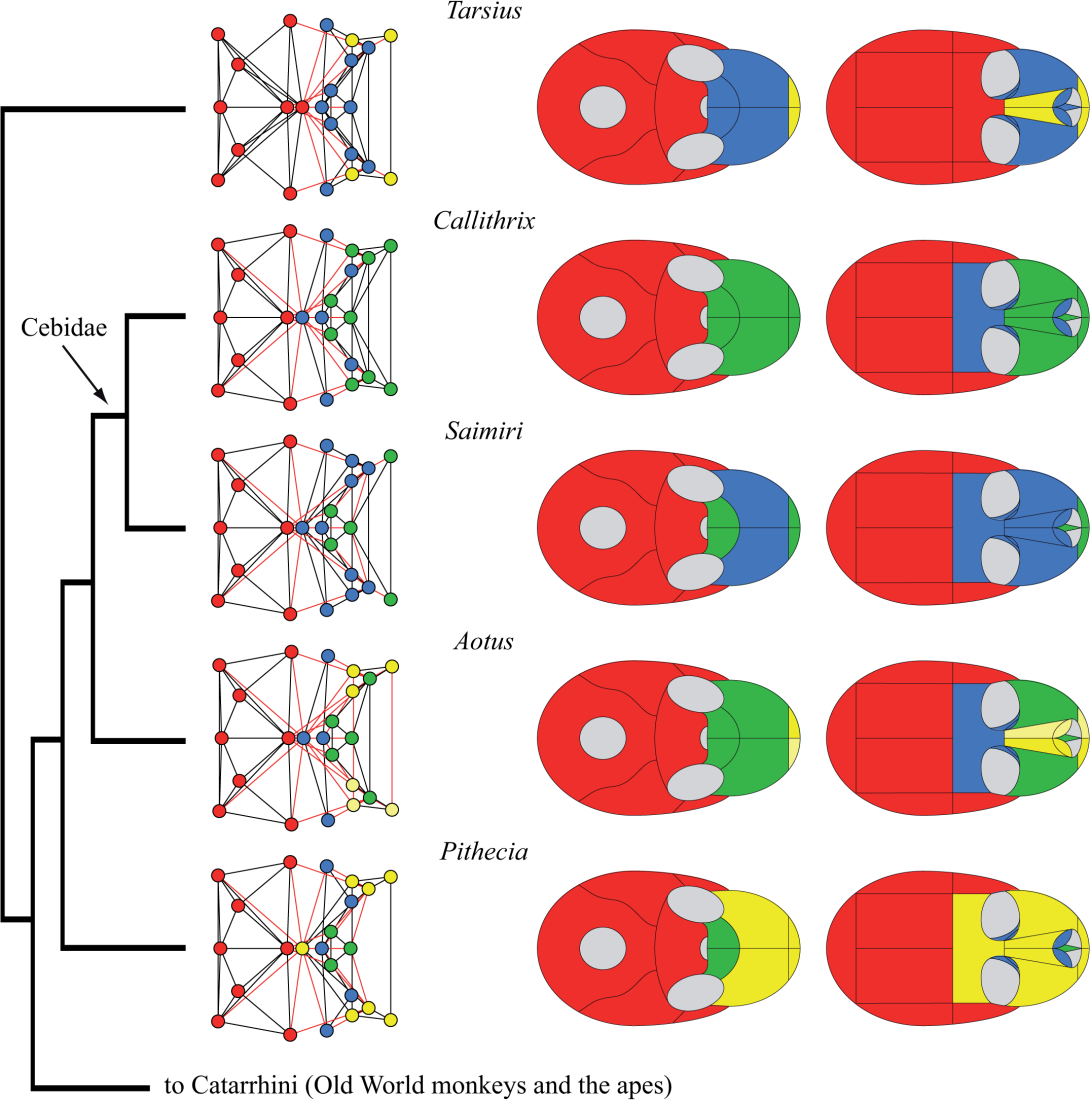
Connectivity modules identified in the skull of Platyrrhini and *Tarsius*. In contrast to Strepsirrhini, the frontal bone is unpaired in the skull of Haplorrhini (but see [33]). In *Tarsius* the frontal bone is included in the cranial module (in *red*), while in Platyrrhini the frontal belongs to the midfacial module (in *blue*) or to the premaxillary module (in *yellow*) in *Colobus*. Platyrrhini (*right*) show a conserved bone composition of the cranial module: occipital, sphenoid, parietals, temporals, and zygomatics. The three types of facial modules are also present, but they vary in their bone composition from one skull to another.

**Fig 5:**
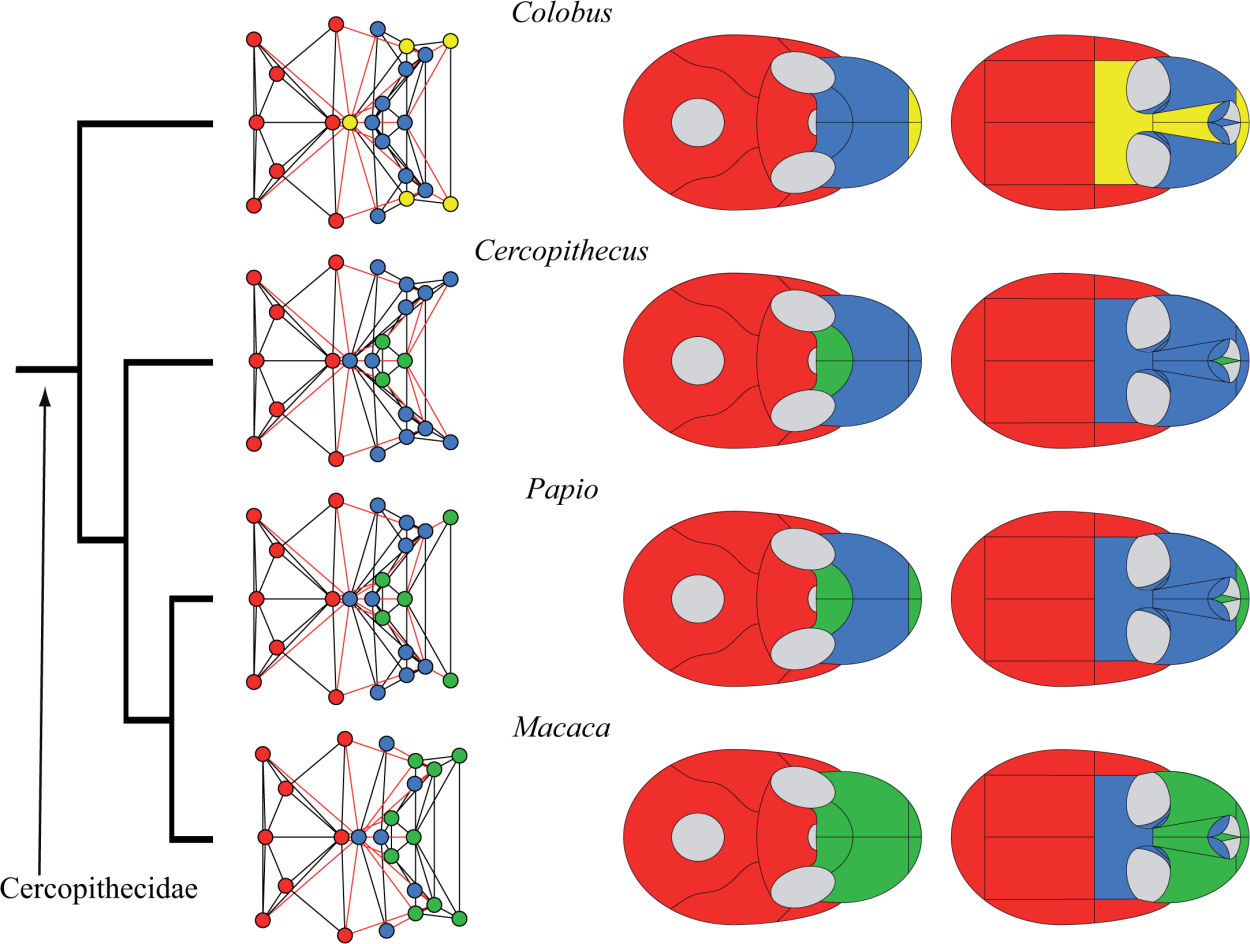
Connectivity modules identified in the skull of Cercopithecidae. Like in Platyrrhini, the skulls of Cercopithecidae (*left*) show a conserved bone composition of the cranial module (in *red*): occipital, sphenoid, parietals, temporals, and zygomatics; as well as variability in the presence and composition of facial modules (in *blue*, *green*, and *yellow*).

**Fig 6:**
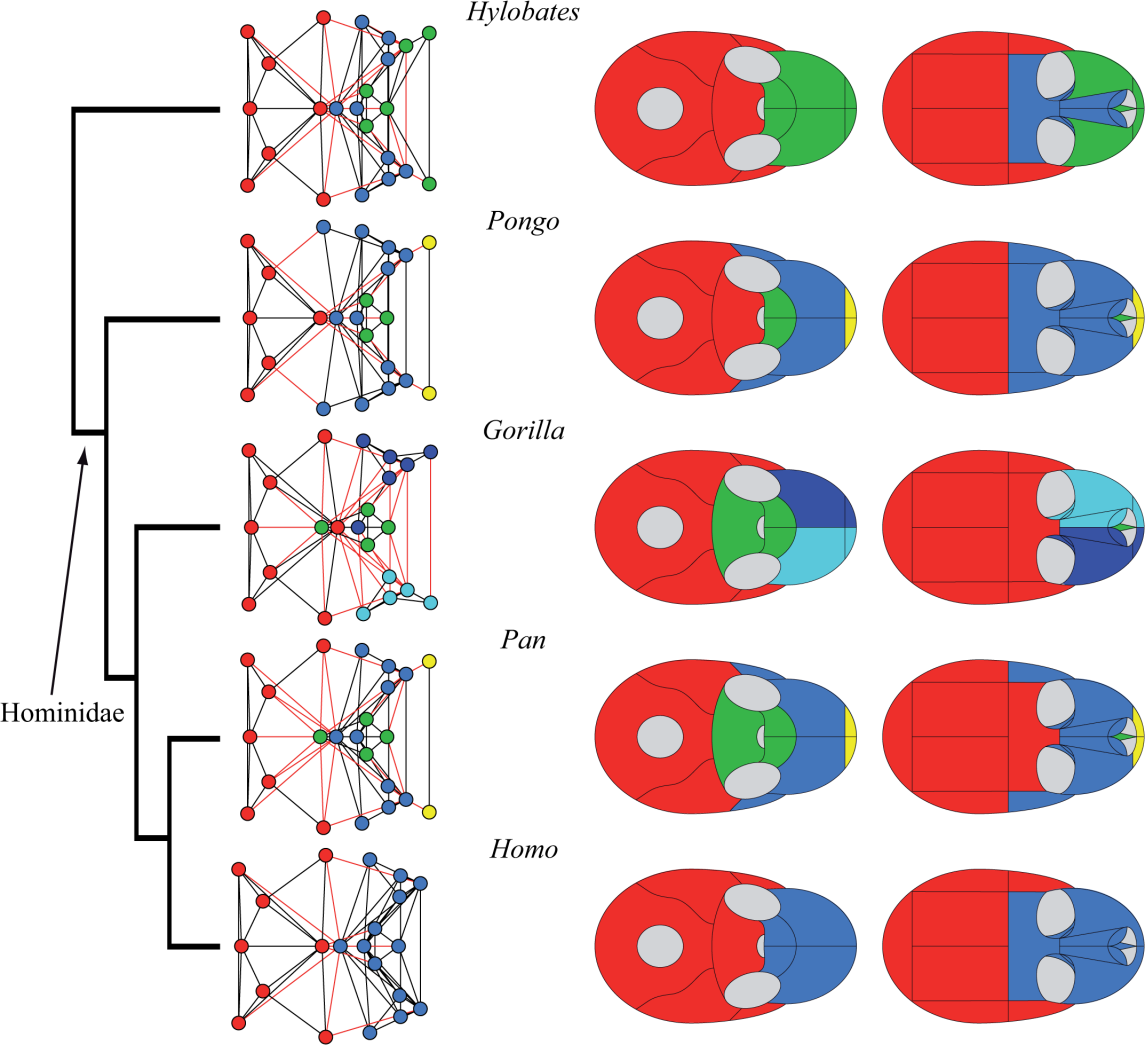
Connectivity modules identified in the skull of Cercopithecidae and Hominoidea. The skulls of Hominoidea (*right*) show a greater variability of bone composition in both the facial and the cranial modules. Within this group we observe the same variability achieved by the other skulls analyzed: one to three facial modules (in *blue*, *green*, and *yellow*), and variation in bones that integrate the cranial module (in *red*). The inclusion of the frontal, sphenoid, and zygomatics in the cranial module varies from one skull to another. The human skull shows a two-module division into facial and cranial modules: a unique organization within Primates, but similar to that of *Tupaia*.

Phenotypic modularity varies among primate skulls in the number of modules (from 2 to 5), in the bones composing these modules, and, as a consequence, in the strength of modularity. Figs 3–6 show the modular organization of the skull of primates in a phylogenetic context.

We found four types of connectivity modules: (1) neurocranial, (2) midfacial, (3) palatal, and (4) premaxillary. The name of each module refers only to the anatomical region it occupies, and excludes any developmental, functional, and evolutionary interpretation. The **neurocranial module** is present in all skull networks and always groups together the occipital, the temporals, and the parietals; often, the neurocranial module also includes the sphenoid (in 18 out of 20 skulls) and/or the zygomatics (15), as well as the frontal/s in *Tarsius* and *Gorilla*, and the non-primate genera *Tupaia* and *Cynocephalus*. The **midfacial module** is present in all skull networks and always groups the ethmoid, the lacrimals, and (except in *Aotus*) the nasal conchae. Sometimes the midfacial module includes the frontal/s (14), the nasals (12), the palatines (6), the vomer (6), and the zygomatics (5). In Strepsirrhini (*Lemur*, *Propithecus*, *Loris*, *Nycticebus*) and in *Gorilla* the midfacial module splits into **left and right midfacial modules**. Here, unpaired bones are always included in the left module, but this is an arbitrary assignment because unpaired bones are equally connected to both sides of the skull. Exceptionally, the left and right palatines are grouped together in the left midfacial module of *Loris* and *Nycticebus*. Consequently, in these two taxa, left and right midfacial modules are asymmetrical because the palatines form a strong cluster that prevents their split in left and right modules. In fact, an independent **palatal module** groups the palatines in 14 out of 20 skull networks, and together with the vomer in 12 of them. The palatal module also includes the premaxillae in *Callithrix*, *Saimiri*, *Macaca*, *Papio*, and *Hylobates*; the maxillae in *Aotus*, *Callithrix*, Macaca, and *Hylobates*; the nasals in *Callithrix* and *Macaca*; and the sphenoid in *Gorilla* and *Pan*. When the palatines do not form an independent module, they form a block within the midfacial module together with the vomer. Finally, an independent **premaxillary module** is present in 9 out of 20 skull networks grouping the premaxillae; often this module includes the nasals (6) and, less frequently, the frontal in *Pithecia* and *Colobus*, the vomer and the maxillae in *Lemur* and *Propithecus*, and the nasal conchae in *Aotus*. In *Aotus* this premaxillary module splits into **left and right premaxillary modules**.

The hierarchical grouping method that we used to identify connectivity modules also allows us to distinguish two main regions (or macromodules) in primate skulls according to the first split of the dendrogram (see SI), which we called facial and cranial because of their anatomical position. The *cranial* macromodule always comprises the neurocranial connectivity module, as well as the palatal module in *Gorilla*, *Pan*, *Pithecia*, and *Pongo*. The *facial* macromodule always comprises the midfacial module and, except for the aforementioned genera, the palatal module. The premaxillary connectivity module is also included within the facial module in all genera except for *Pan* and *Tarsius*, whose premaxillary module splits in the first place from both macromodules. Thus, we observe distinct palatal and premaxillary modules in the skull of primates in addition to the previously reported facial and cranial modules.

### The near-decomposability hypothesis

The skulls of the genera analyzed show a narrow range of variation in complexity and modularity (Table 1). The Abouheif's test indicates a significant phylogenetic signal in network parameters measuring complexity as number of parts (N: *p* = 0.003; K: *p* = 0.019). In contrast, network parameters measuring complexity as richness of interaction do not show a phylogenetic signal (D: *p* = 0.809; C: *p* = 0.066; H: *p* = 0.424). Interestingly, modularity lacks also of a phylogenetic signal in primates (M: *p* = 0.192).

**Table 1:** Network parameters quantifying modularity and complexity.

	Min.	1^st^ Quartile	Median	Mean	3^rd^ Quartile	Max.
**Modularity (M)**	0.5486	0.9074	0.9815	1.06	1.253	1.6368
**Bones (N)**	21	23	23	23.2	24	24
**Interactions (K)**	60	61.75	63.5	64.3	67.25	74
**Compactness (D)**	0.2174	0.2411	0.2464	0.2502	0.2569	0.3048
**Redundancy (C)**	0.3589	0.4877	0.5326	0.5266	0.5828	0.6339
**Disparity (H)**	0.4625	0.4901	0.5059	0.5185	0.5389	0.6689

Pearson’s product-moment correlations show a significant positive correlation between the modularity and the complexity measured as the number of bones (N: *r* = 0.691, *p* = 5.28e^–4^), as predicted by the near-decomposability hypothesis. However, the other parameters used as measures of complexity lack this positive correlation with modularity; instead we observe a negative correlation between modularity and complexity (K: *r* = –0.442, *p* = 0.029; D: *r* = –0.701, *p* = 4.12e^–4^; C: *r* = –0.409, *p* = 0.041). Finally, disparity or anisomerism does not correlate at all with modularity (H: *r* = 0.149, *p* = 0.729).

These results do not support the existence of a positive feedback loop between modularity and complexity during primate skull evolution, when we measured complexity as richness of interactions. Conversely, if we limit our definition of complexity to the number of bones, then our results support a positive feedback loop between modularity and complexity in this study; however, we view this result with some caution because of the narrow range of variation in the number of skull bones among primates.

## Discussion

Our phylogenetic comparison of modularity in primates suggests a division of the skull into two phenotypic modules: one facial and one cranial. Previous studies have also identified these two regions as two separate connectivity modules in the adult human skull [27] and in newborn human skulls with various craniosynostosis conditions [28]. The division observed resembles the classic division of the mammalian skull into viscerocranium and neurocranium [34], although connectivity modules delimit different boundaries for these two regions. In primate skulls, we observe greater variability in the connectivity modules that form the facial module than in those that form the cranial module. For example, the facial region comprises up to three connectivity modules (midfacial, palatal, and premaxillary), which can be subsumed one into another, split in left and right specular modules, and/or group different bones. In contrast, the cranial region comprises invariably the neurocranial connectivity module, plus the palatal connectivity module in *Gorilla*, *Pan*, *Pithecia*, and *Pongo*. Phylogenetically, it is more parsimonious to postulate that this feature was acquired in great apes and then lost in humans (two steps; in addition to the independent acquisition in *Pithecia*) than that it was acquired in *Gorilla*, *Pan* and *Pongo* independently (3 steps). Therefore, this constitutes a synapomorphy of the Hominidae (*Pongo*, *Gorilla*, *Pan* and *Homo*), and might thus indicate that anatomical networks can also be useful for taxonomic and phylogenetic studies. The evolutionary and ecomorphological implications of this feature need to be studied in future works. In general, the cranial region shows only minor variations between major phylogenetic groups, such as Strepsirrhini, Platyrrhini, and Cercopithecidae (Figs 3 to 5). Exceptionally within Hominidae (*Pongo*, *Gorilla*, *Pan* and *Homo*), the cranial region varies in how it groups the frontal, sphenoid, and zygomatic bones (Fig 6). Previous studies showed that these bones act specifically connecting (i.e. integrating) the facial and the cranial regions in humans [27].

Connectivity modules derive from the analysis of the overall pattern of bone contacts in a skull, but they capture a deep organization of the growth patterns of the skull because bone contacts (i.e. craniofacial sutures) are primary sites of bone growth. As a consequence, the facial and cranial phenotypic modules identified have further developmental, variational, and functional implications for understanding the evolution of the human head [23]. Notably, the phenotypic modules defined in the present study discriminate the sutures of the skull according to the sequence of closure proposed for hominoids [35]. The cranial module includes the sutures that close earlier (vault, base, and circummeatal regions), whereas the facial region includes the sutures that close later (palatal, facial, and craniofacial regions). Interestingly, the patterns of sutures in the cranial region are conserved even in species as distantly related to humans as zebrafish [36]. This invariance of the suture patterns in the cranial region is surprising in the context of the tremendous increase in brain size, and associated changes in facial shape, during the evolution of primates [37], and suggests a deep conservation of skull morphogenesis across tetrapods.

Characterizing growth processes is essential to understand craniofacial shape variation during the evolution and development of the skull [4]. Thus, in addition to behaving as different units of allometric growth in humans [27], we would expect connectivity modules to be consistent with reports based on phenotypic variation in the size and shape of the skull. Our finding of a unitary cranial module agrees with the presence of an integrated cranial module (vault and base) in humans that shows patterns of covariation during postnatal ontogeny [38]. Along similar lines, a recent study by Villmoare et al. [39] has also reported the presence of premaxillary and palatal modules in the face of extant primates and extinct human relatives, similarly to what we found in this study analyzing connectivity patterns. Other studies have found that indeed the facial and cranial regions vary also in the degree of intra-module integration, which directly affects shape co-variation, functional coordination, and responses to selective pressures [6,40,41]. Moreover, Goswami and Polly [42] have demonstrated that the facial modules differ from cranial modules in their evolutionary plastic and morphological disparity in Primates. As a consequence, any change in the connectivity patterns of the skull, for example a fusion of frontal bones, might provoke specific shape changes in the entire skull. The premature fusion of bones (i.e. craniosynostosis) is a striking example of how a change in a single bone contact affects the shape of the skull, as a consequence of compensatory growth in its vicinity as well as in more distant parts of the skull [43–45].

Although deeply conserved developmental processes are likely to be responsible for much of the observed modularity in organisms, Wagner [20] argued that changes in modularity, particularly an increase in modularity, is likely to be the result of selection. Modularity allows more rapid change, and phenotypes that can change more rapidly in response to changing selection pressures have an adaptive advantage. Differences in modularity among primates are likely to be adaptive responses to differing selective pressures. In Platyrrhini, Marroig and Cheverud [46] found that patterns of morphological integration, while clearly influenced by phylogeny, also closely matched shared dietary patterns, a result supported by Makedonska and co-workers [47]. One result of our analysis (Fig 3) shows that, while conserved patterns are apparent in Strepsirrhini, lemurs have acquired a derived pattern. This is consistent with the relatively derived behavioral patterns seen in this clade. Similarly, in our results, within Catarrhinii (Fig 5), *Colobus* shows a relatively derived pattern of cranial modularity, which suggests that its unique dietary adaptation may influence patterns of modularity. The unique loss of the premaxilla in *Homo*, and its incorporation within the maxilla, is likely linked to adaptive changes relating to the anterior dentition. Overall, the conserved modularity of the neurocranium compared to the variable patterns of facial modularity seems to support adaptive causes for facial evolution in primates.

As Klingenberg [48] notes, however, changes in modularity and integration will alter the evolutionary constraints of a lineage. Certain phenotypes will no longer be accessible if modularity biases the directions of response to selection. Questions of evolvability and constraint are central to understanding how the varying influences of integration, modularity, and ontogeny interact under the pressure of natural selection to produce the resultant phenotype [49]. Biasing the directions in which responses to selection may operate—by changing the patterns of connectivity in the cranial skeleton—can have strongly advantageous consequences, if adaptive peaks in the fitness landscape fall along the available trajectories [50]. But if integration or modularity constrains the available variation, and peaks cannot be reached, organisms may not be able to respond before becoming extinct [51,52]. Primates are noteworthy for their adaptive diversity, and in future research we intend to investigate the relationship between adaptive diversity, morphological disparity, and modular variation, to determine if modular constraints are at play in other, less diverse clades, which are not present in Primates.

In conclusion, our results showed that the number of modules does not correlate positively with our complexity measures. In Simon’s view, a system (here, the skull) is organized hierarchically when it is composed of interrelated subsystem (the modules) that are nested until reaching some lowest level of elementary subsystems (the bones). Taking number of modules as a proxy for the hierarchical arrangement of the skull (more modules = more hierarchy), Simon’s *near-decomposability hypothesis* does not hold for the primate data. Instead, what we observed in Primates is an inverse relationship between these two properties: more complex skulls show lower modularity. This suggests that in primates the formation of new bone contacts, which foster complexity after fusion and loss of bones [30], also integrates modules and lessens the parcellation of the skull. However, we cannot neglect that the narrow range of values of the parameters used to quantify complexity in the skull of primates, if we compare it with the values of tetrapods [29], might conceal this correlation. A promising way of testing and further expanding the findings of this study is to analyze taxonomic groups of higher rank and with greater skull disparity such as the whole Tetrapoda clade. Our study paves the way to undertake such studies, as well as test and discuss other related hypotheses seeking to understand the evolution of phenotypic modularity and complexity in vertebrates.

## Materials and Methods

### Skull data and phylogeny

We studied the skull of 20 Euarchonta taxa: 18 primate taxa representing all the major extant groups of primates, and representatives of the two closest living relatives of primates: Dermoptera (*Cynocephalus*) and Scandentia (*Tupaia*) [53]. We coded for articulations among bones to model skull networks by inspecting natural skulls and casts housed in the zoology collection of the Dept. of Archaeology, Univ. of Saskatchewan. Data were collected from the following specimens: *Aotus* (043, 044), *Callithrix* (024, 025), *Cercopithecus* (Z-001), *Colobus* (Z-010), *Cynocephalus* (e-resources see below), *Gorilla* (24–8799, 081, 083, 085–90), *Homo* (e-resources, see below), *Hylobates* (24–8779), *Lemur* (BC-087, 022), *Loris* (019, 020), *Macaca* (POW-01-12, POW-01-13, 24–8696), *Nycticebus* (021), *Pan* (BC-003, 24–8794, 24–8792), *Papio* (063, 24–8744, 24–8745), *Pithecia* (e-resources, see below), Pongo (P-761, 24–8788, 078, 079), Propithecus (BC-284, 023), Saimiri (24–8622, 24–8623, PNW-03-12), Tarsius (017, 018), Tupaia (001, 003). No permits were required for the described study, which complied with all relevant regulations. We supplemented the study of actual skulls using online resources: Digimorph (digimorph.org), Univ. of Michigan Animal Diversity Web (animaldiversity.ummz.umich.edu), Univ. of Texas (Austin) E-Skeletons (eskeletons.org/index.html), Will’s Skull Page (skullsite.co.uk/index.htm); as well as Hill’s series of books, *Primates*: *Comparative Anatomy and Taxonomy* (1953–1974). We calibrated the phylogeny of primates according to molecular estimations [32]; branch lengths indicate time of divergence in millions of years.

### Modeling anatomical networks

Bone-bone articulations were coded in three-dimensional unweighted network models, in which network nodes represent skull bones and connections represent craniofacial sutures and synchondroses. For each skull, we coded 1/0 for the presence/absence of contact between two bones in a symmetric adjacency matrix of size equal to bone number. We performed the modeling and analysis of skull networks in *R* (R Core Team 2014) using the package *igraph* [54]. Adjacency matrices of skulls are available at http://dx.doi.org/10.6084/m9.figshare.1221540. The protocol used to analyze each skull network is detailed in the S1 Protocol.

### Identifying connectivity modules in anatomical networks

A connectivity module in a skull network is a group of bones with more contacts among them than to other bones outside the module [27]. We identified the number and bone composition of connectivity modules using a hierarchical cluster analysis of the pair-wise bone similarity, which is quantified as the generalized topological overlap (TO) between each pair of bones. TO is a normalized measure of similarity that quantifies pair-wise common neighbors between the nodes of a network: *TO*_*i*,*j*_ = *TO*_*j*,*i*_ = *J*(*n*_*i*_, *n*_*j*_)/min_*k*_(*i*, *j*), where *J(n*_*i*_,*n*_*j*_ *)* is the total amount of neighbors in common between two nodes and *min*_*k*_ *(i*,*j)* is the lowest connectivity of both nodes. TO ranges from 0 to 1: two nodes that connect to all the same other nodes have TO = 1, whereas two nodes without any neighboring node in common have TO = 0. This similarity index has been extensively used to analyze modularity in different types of networks [55,56]. Hierarchical clustering gathers together bones with higher TO into single branches until all bones form one group. For each potential partition we quantified Q: a quality index that quantifies how well a potential partition groups the nodes of the network compared to other possible partitions [57]: $Q=\frac{1}{2k}\sum_{i,j}\left[A_{ij}-\frac{k_{i}\times k_{j}}{2k}\right]\times \delta \left(m_{i},m_{j}\right)$, where *K* is the number of connections, *A*_*ij*_ is the adjacency matrix, *k*_*i*_ is the connections of *i*, *k*_*j*_ that of *j*, *m*_*i*_ is the module of *i*, and *m*_*j*_ that of *j*. If *m*_*i*_ = *m*_*j*_ then *δ(m*_*i*_,*m*_*j*_ *)* = 1, else *δ (m*_*i*_,*m*_*j*_ *)* = 0. If the number of connections among nodes in the same module is not higher than expected at random then Q = 0, otherwise Q > 0: the higher the Q, the better the partition. For each skull network, the partition with the highest Q identifies its connectivity modules. We quantified the modularity of the skull network (M) as the product of the number of modules and Q. We identified connectivity modules using the functions hclust (package *stats*), GTOMmdist1 (labs.genetics.ucla.edu/horvath/GTOM/old/gtom.R), and modularity, package *igraph*.

### Estimating complexity in anatomical networks

We estimated the complexity of skull using five network parameters: number of nodes (N), number of connections (K), density of connections (D), average clustering coefficient (C), and heterogeneity of connections (H). We chose these parameters as proxies to estimate diverse features that have been associated in the past with the morphological complexity of the skull [29].

N and K are the basic descriptors of the network: the number of nodes and the number of links. D is the number of existing node-node connections with respect to the total maximum possible according to the total number of nodes: *D* = 2*k*/*N*(*N* − 1). C is the average of the clustering coefficients of all nodes in the network: $C=\frac{1}{N}\sum \left(\sum \tau_{i}/\sum \left(k_{i}/\left(k_{i}-1\right)\right)\right)$, where *τ*_*i*_ is the number of triangular motifs that include node *i* and *k*_*i*_ is the number of connections of node *i*. H is the ratio between the variance and the mean of connectivity: *H* = *σ*_*K*_/*μ*_*K*_, where *σ*_*K*_ is the variance of the number of connections of all nodes in the network and *μ*_*K*_ is the mean of the number of connections.

### Estimating the phylogenetic signal of modularity and complexity

We tested the phylogenetic signal of the parameters estimating modularity (M) and complexity (N, K, D, C, H) using the Abouheif’s test with 1000 permutations [58]. A significant phylogenetic signal would indicate a phylogenetically constrained evolution of modularity and/or complexity in Primates. We estimated the phylogenetic signal with the function abouheif.moran, package *phytools* [59]. We also performed a Blomberg’s test (see SI).

### Testing the near-decomposability hypothesis

According to Simon’s *near-decomposability hypothesis* modularity and complexity evolve in a positive feedback. We tested the null hypothesis that there is no correlation between modularity (M) and complexity (N, K, D, C, H). Our alternative hypothesis was that modularity and complexity are positively correlated. We performed Pearson’s product-moment correlations of phylogenetic independent contrasts using the method FIC4 [60]. We performed these tests with the function pic, package *ape* [61], and the function cor.test (package *stats*).

## Supporting Information

S1 Protocol R Markdown file used to analyze the data. (HTML)

## References

[1] Lieberman DE (2011) The evolution of the human head London: Belknap Press.

[2] Ross CF (2013) Complexity, Modularity, and Integration in the Human Head. The Evolution of the Human Head. J Hum Evol 64: 56–67.

[3] Adams DC, Rohlf FJ, Slice DE (2013) A field comes of age: Geometric morphometrics in the 21st century. Hystrix, It J Mamm 24: 7–14.

[4] Martínez-Abadías N, Mitteroecker P, Parsons TE, Esparza M, Sjovold T, et al (2012) The developmental basis of quantitative craniofacial variation in humans and mice. Evol Biol 39: 554–567. 23226904 10.1007/s11692-012-9210-7 PMC3514712

[5] Singh N, Harvati K, Hublin JJ, Klingenberg CP (2012) Morphological evolution through integration: a quantitative study of cranial integration in Homo, Pan, Gorilla and Pongo. J Hum Evol 62: 155–164. 10.1016/j.jhevol.2011.11.006 22178399

[6] Porto A, Shirai LT, de Oliveira FB, Marroig G (2013) Size variation, growth strategies, and the evolution of modularity in the mammalian skull. Evolution 67: 3305–3322. 10.1111/evo.12177 24152009

[7] Bonner JT (1988) The evolution of complexity by means of natural selection Princeton: Princeton University Press.

[8] McShea DW (1993) Evolutionary change in the morphological complexity of the mammalian vertebral column. Evolution 47: 730–730. 28567892 10.1111/j.1558-5646.1993.tb01229.x

[9] Diogo R, Ziermann JM, Linde-Medina M (2014) Is evolutionary biology becoming too politically correct? A reflection on the scalae naturae, phylogenetically basal clades, anatomically plesiomorphic taxa, and "lower" animals. Biol Rev. 10.1111/brv.12121 24917249

[10] Diogo R, Ziermann JM, Linde-Medina M (2014) Specialize or risk disappearance—empirical evidence of anisomerism based on comparative and developmental studies of gnathostome head and limb musculature. Biol Rev. 10.1111/brv.12142 25174804

[11] Gregory WK (1935) ‘Williston's law’ relating to the evolution of skull bones in the vertebrates. Am J Phys Anthropol 20: 123–152.

[12] Sidor CA (2001) Simplification as a trend in synapsid cranial evolution. Evolution 55: 1419–1442. 11525465 10.1111/j.0014-3820.2001.tb00663.x

[13] McShea DW, Hordijk W (2013) Complexity by subtraction. Evol Biol 40: 504–520.

[14] Simon HA (1962) The architecture of complexity. Proc Amer Phil Soc 106: 467–482.

[15] Simon HA (2005) The structure of complexity in an evolving world: the role of near decomposability In: Callebaut W, Rasskin-Gutman D, editors. Modularity: Understanding the development and evolution of natural complex systems. Cambridge: The MIT Press pp. ix–xiii.

[16] Raff RA (1996) The shape of life: Genes, development, and the evolution of animal form Chicago: The University of Chicago Press.

[17] Schlosser G, Wagner GP (2004) Modularity in development and evolution Chicago: University of Chicago Press.

[18] Callebaut W, Rasskin-Gutman D, editors (2005) Modularity: Understanding the development and evolution of natural complex systems Cambridge: MIT press.

[19] West-Eberhard MJ (2003) Developmental plasticity and evolution Oxford: Oxford University Press.

[20] Wagner GP (1996) Homologues, natural kinds and the evolution of modularity. Am Zool 36: 36–43.

[21] Brigandt I (2007) Typology now: homology and developmental constraints explain evolvability. Biol Phil 22: 709–725.

[22] Rasskin-Gutman D, Esteve-Altava B (2014) Connecting the Dots: Anatomical Network Analysis in Morphological EvoDevo. Biol Theory 9: 178–193.

[23] Esteve-Altava B, Diogo R, Smith C, Boughner JC, Rasskin-Gutman D (2015) Anatomical networks reveal the musculoskeletal modularity of the human head. Sci Rep 5: 8298 10.1038/srep08298 25656958 PMC5389032

[24] Opperman LA (2000) Cranial sutures as intramembranous bone growth sites. Dev Dyn 219: 472–485. 11084647 10.1002/1097-0177(2000)9999:9999<::AID-DVDY1073>3.0.CO;2-F

[25] Herring SW (2008) Mechanical influences on suture development and patency In: Rice DP, editor. Craniofacial sutures, development, disease and treatment. London: Karger pp. 41–56. 10.1159/0000115031 PMC2826139 18391494

[26] Di Ieva A, Bruner E, Davidson J, Pisano P, Haider T, et al (2013) Cranial sutures: A multidisciplinary review. Childs Nerv Syst 29: 893–905. 10.1007/s00381-013-2061-4 23471493

[27] Esteve-Altava B, Marugan-Lobon J, Botella H, Bastir M, Rasskin-Gutman D (2013) Grist for Riedl's mill: a network model perspective on the integration and modularity of the human skull. J Exp Zool Part B 320: 489–500. 10.1002/jez.b.22524 23913546

[28] Esteve-Altava B, Rasskin-Gutman D (2015) Evo-Devo insights from pathological networks: Exploring craniosynostosis as a developmental mechanism for modularity and complexity in the human skull. J Anthropol Sci 93: 1–15. 25324462 10.4436/JASS.93001

[29] Esteve-Altava B, Marugán-Lobón J, Botella H, Rasskin-Gutman D (2013) Structural constraints in the evolution of the tetrapod skull complexity: Williston’s law revisited using network models. Evol Biol 40: 209–219.

[30] Esteve-Altava B, Marugán-Lobón J, Botella H, Rasskin-Gutman D (2014) Random loss and selective fusion of bones originate morphological complexity trends in tetrapod skull networks. Evol Biol 41: 52–61.

[31] Gregory WK (1934) Polyisomerism and anisomerism in cranial and dental evolution among vertebrates. Proc Natl Acad Sci U S A 20: 1–9. 16587830 10.1073/pnas.20.1.1 PMC1076327

[32] Perelman P, Johnson WE, Roos C, Seuanez HN, Horvath JE, et al (2011) A molecular phylogeny of living primates. PLoS Genet 7: e1001342 10.1371/journal.pgen.1001342 21436896 PMC3060065

[33] Rosenberger AL, Pagano AS (2008) Frontal fusion: Collapse of another anthropoid synapomorphy. Anat Rec 291: 308–317. 10.1002/ar.20647 18231970

[34] Moore WJ (1981) The Mammalian Skull. Cambridge: Cambridge University Press.

[35] Krogman WM (1930) Studies in growth changes in the skull and face of anthropoids. II. Ectocranial and endocranial suture closure in anthropoids and Old World Apes. Am J Anat 46: 315–353.

[36] Quarto N, Longaker MT (2008) The zebrafish (*Danio rerio*): A model system for cranial suture patterning. Cells Tissues Organs 181: 109–118. 10.1159/000091100 16534205

[37] Bastir M, Rosas A, Gunz P, Pena-Melian A, Manzi G, et al (2011) Evolution of the base of the brain in highly encephalized human species. Nat Commun 2: 588 10.1038/ncomms1593 22158443

[38] Barbeito-Andrés J, Sardi ML, Anzelmo M, Pucciarelli HM (2012) Functional matrices and morphological integration. An ontogenetic study on the vault and the maxilla. Revista Argentina de Antropología Biológica 14: 79–87.

[39] Villmoare BA, Dunmore C, Kilpatrick S, Oertelt N, Depew MJ, et al. (2014) Craniofacial modularity, character analysis, and the evolution of the premaxilla in early African hominins. J Hum Evol. 10.1016/j.jhevol.2014.06.014 25449953

[40] Porto A, de Oliveira FB, Shirai LT, De Conto V, Marroig G (2009) The evolution of modularity in the mammalian skull I: morphological integration patterns and magnitudes. Evol Biol 36: 118–135.

[41] Marroig G, Shirai LT, Porto A, de Oliveira FB, De Conto V (2009) The evolution of modularity in the mammalian skull II: Evolutionary consequences. Evol Biol 36: 136–148.

[42] Goswami A, Polly PD (2010) The influence of modularity on cranial morphological disparity in Carnivora and Primates (Mammalia). PLoS One 5: e9517 10.1371/journal.pone.0009517 20209089 PMC2831076

[43] Heuzé Y, Boyadjiev SA, Marsh JL, Kane AA, Cherkez E, et al (2010) New insights into the relationship between suture closure and craniofacial dysmorphology in sagittal nonsyndromic craniosynostosis. J Anat 217: 85–96. 10.1111/j.1469-7580.2010.01258.x 20572900 PMC2913018

[44] Heuzé Y, Martínez-Abadías N, Stella JM, Senders CW, Boyadjiev SA, et al (2012) Unilateral and bilateral expression of a quantitative trait: asymmetry and symmetry in coronal craniosynostosis. J Exp Zool Part B 318: 109–122. 10.1002/jezb.21449 22532473 PMC3315613

[45] Martinez-Abadias N, Heuze Y, Wang Y, Jabs EW, Aldridge K, et al (2011) FGF/FGFR signaling coordinates skull development by modulating magnitude of morphological integration: evidence from Apert syndrome mouse models. PLoS One 6: e26425 10.1371/journal.pone.0026425 22053191 PMC3203899

[46] Marroig G, Cheverud JM (2001) A comparison of phenotypic variation and covariation patterns and the role of phylogeny, ecology, and ontogeny during cranial evolution of new world monkeys. Evolution 55: 2576–2600. 11831671 10.1111/j.0014-3820.2001.tb00770.x

[47] Makedonska J, Wright BW, Strait DS (2012) The effect of dietary adaption on cranial morphological integration in capuchins (order Primates, genus Cebus). PLoS One 7: e40398 10.1371/journal.pone.0040398 23110039 PMC3482247

[48] Klingenberg CP (2005) Developmental constraints, modules and evolvability In: Hallgrímsson B, Hall BK, editors. Variation. San Diego: Academic Press pp. 219–247.

[49] Hendrikse JL, Parsons TE, Hallgrımsson B (2007) Evolvability as the proper focus of evolutionary developmental biology. Evol Dev 9: 393–401. 17651363 10.1111/j.1525-142X.2007.00176.x

[50] Hansen TF (2003) Is modularity necessary for evolvability? Remarks on the relationship between pleiotropy and evolvability. Biosystems 69: 83–94. 12689723 10.1016/s0303-2647(02)00132-6

[51] Orr H, Unkless R (2008) Population extinction and the genetics of adaptation. Am Nat 172: 160–169. 10.1086/589460 18662122

[52] Gomulkiewicz R, Houle D (2009) Demographic and genetic constraints on evolution. Am Nat 174: 218–229. 10.1086/645086 19821744

[53] Diogo R, Wood B (2012) Comparative anatomy and phylogeny of primate muscles and human evolution Oxford: Tayloer & Francis.

[54] Csardi G, Nepusz T (2006) The igraph software package for complex network research. Int J Comp Syst Sci 1695.

[55] Ravasz E, Somera AL, Mongru DA, Oltvai ZN, Barabási A-L (2002) Hierarchical organization of modularity in metabolic networks. Science 297: 1551–1555. 12202830 10.1126/science.1073374

[56] Yip AM, Horvath S (2007) Gene network interconnectedness and the generalized topological overlap measure. BMC Bioinformatics 8: 22 17250769 10.1186/1471-2105-8-22 PMC1797055

[57] Newman ME, Girvan M (2004) Finding and evaluating community structure in networks. Phys Rev E 69: 026113 14995526 10.1103/PhysRevE.69.026113

[58] Abouheif E (1999) A method for testing the assumption of phylogenetic independence in comparative data. Evol Ecol Res 1: 895–909.

[59] Revell LJ (2013) Two new graphical methods for mapping trait evolution on phylogenies. Met Ecol Evol 4: 754–759.

[60] Laurin M (2010) Assessment of the relative merits of a few methods to detect evolutionary trends. Syst Biol 59: 689–704. 10.1093/sysbio/syq059 20937759

[61] Paradis E, Claude J, Strimmer K (2004) APE: analyses of phylogenetics and evolution in R language. Bioinformatics 20: 289–290. 14734327 10.1093/bioinformatics/btg412

