# Supplementary material for: Anatomical Network Analysis Shows Decoupling of Modular Lability and Complexity in the Evolution of the Primate Skull
Source: PLoS One. 2015 May 19;10(5):e0127653. doi: 10.1371/journal.pone.0127653 (PMC4438065; doi:10.1371/journal.pone.0127653)
Supplement: S1 Protocol — (HTML) [file pone.0127653.s001.html]

## Supporting Information

### Anatomical network analysis shows decoupling of modular lability and complexity in the evolution of the primate skull

Borja Esteve-Altava, Julia C. Boughner, Rui Diogo, Brian A. Villmoare, and Diego Rasskin-Gutman

**Data location**: figshare.com/s/1cc7039aba8311e4ba3006ec4b8d1f61 (http://dx.doi.org/10.6084/m9.figshare.1221540)

**Description**: Protocol of the anatomical network
analysis performed to analyze modularity and complexity in the 20 skull
network models sudied.

##### 0. Content

1. Pre-processing skull networks
2. Identifying connectivity modules in skull networks
3. Quantifying the modularity and complexity using network parameters
4. Estimating the phylogenetic signal of network parameters
5. Testing the Near-decomposability Hypothesis

##### 1. Pre-processing skull networks

We load the raw adjacency matrices from the repository and convert them into igraph objects.

```
  # load data https://github.com/Boresal/Primates/blob/master/primateAdjm.xls?raw=true
mat.list <- mapply(read.xls, xls = "primateAdjm.xls", sheet=1:20)
species <- c("Aotus", "Callithrix", "Cercopithecus", "Colobus", "Cynocephalus", "Gorilla", "Homo", "Hylobates", "Lemur", "Loris", "Macaca", "Nycticebus", "Pan", "Papio", "Pithecia", "Pongo", "Propithecus", "Saimiri", "Tarsius", "Tupaia")
parameter.names <- c("Bones (N)", "Interactions (K)", "Compactness (D)", "Redundancy (C)", "Disparity (H)", "Modularity (M)")
names(mat.list) <- species
  # create adjacency matrices
adj.list <- lapply(mat.list, data.matrix, rownames.force = TRUE)
  # create igraph objects
graph.list <- lapply(adj.list, graph.adjacency, mode = "undirected")
```

##### 2. Identifying connectivity modules in skull networks

We identify connectivity modules using a hierarchical cluster
analysis of the topological overlap matrix, which is used as the
dissimilarity matrix.

```
  # compute topological overlap
  source("http://labs.genetics.ucla.edu/horvath/GTOM/old/gtom.R") # load GTOMmdist1()
tom.list <- lapply(adj.list, GTOMmdist1, m = 1)
  # compute hierarchical cluster analysis
dist.tom.list <- lapply(tom.list, as.dist)
hclust.list <- lapply(dist.tom.list, hclust, method = "average") # method UPGMA
for (i in 1:20){hclust.list[[i]]$labels <- V(graph.list[[i]])$name}
  # extract membership, max. q value, and number of modules (best split) 
membership.list <- list(); q.value.vector <- vector(); split.vector <- vector()
for (i in 1:20){
        nodes <- vcount(graph.list[[i]])
        q.value <- vector()
        for (j in 1:nodes){
                membership <- as.vector(cutree(hclust.list[[i]], k = j))
                q.value[j] <- modularity(graph.list[[i]], membership)
        }
        split.vector[i] <- which(q.value == max(q.value)) # best partition
        membership.list[i] <- list(as.vector(cutree(hclust.list[[i]], k = split.vector[i])))
        q.value.vector[i] <- max(q.value)
}
```

A dendrogram summarizes the identified modules using a different
color for each of the connectivity modules. Dendrograms show the
hierarchical grouping of bones into modules.

##### 3. Quantifying the modularity and complexity using network parameters

We quantify the modularity of skull networks (M) as the product of
the number of modules and the quality of the partition (estimated as the
max. Q value).

We quantify the complexity of each skull network using the following
network parameters: - Number of nodes (N): a proxy for the number of
bones - Number of links (K): a proxy for the number of interactions -
Density of connections (D): a proxy for skull compactness - Clustering
coefficient (C): a proxy for bone contacts redundancy - Heterogeneity of
connections (H): a proxy for skull bones disparity or diversity

```
  # compute network parameters
net.complexity = matrix(0, ncol = 6, nrow = 20)
colnames(net.complexity) <- parameter.names
rownames(net.complexity) <- species
for (i in 1:20){
        net.complexity[i,1] <- vcount(graph.list[[i]])
        net.complexity[i,2] <- ecount(graph.list[[i]])
        net.complexity[i,3] <- graph.density(graph.list[[i]])
        net.complexity[i,4] <- transitivity(graph.list[[i]], type="average", isolates="zero")
        net.complexity[i,5] <- sqrt(var(degree(graph.list[[i]])))/mean(degree(graph.list[[i]]))
        net.complexity[i,6] <- split.vector[i]*q.value.vector[i]
}
print(net.complexity)
```

```
##               Bones (N) Interactions (K) Compactness (D) Redundancy (C) Disparity (H) Modularity (M)
## Aotus                23               63       0.2490119      0.4889014     0.4918714      1.4814815
## Callithrix           23               63       0.2490119      0.4841082     0.5129733      0.9988662
## Cercopithecus        23               62       0.2450593      0.4900469     0.5696146      0.8939906
## Colobus              23               67       0.2648221      0.6018445     0.5114082      0.9118957
## Cynocephalus         24               68       0.2463768      0.5426467     0.4673804      1.2214533
## Gorilla              23               64       0.2529644      0.5610496     0.5358569      1.2504883
## Homo                 21               64       0.3047619      0.6338661     0.4946553      0.5485840
## Hylobates            23               69       0.2727273      0.5764236     0.4846117      0.9770006
## Lemur                24               62       0.2246377      0.3588624     0.4625015      1.4418574
## Loris                24               68       0.2463768      0.5226070     0.4927639      0.8690528
## Macaca               23               61       0.2411067      0.4611833     0.5345437      0.9703037
## Nycticebus           24               68       0.2463768      0.5226070     0.4927639      0.8690528
## Pan                  23               60       0.2371542      0.6140266     0.6688553      1.0216667
## Papio                23               65       0.2569170      0.6169524     0.5660752      0.9418935
## Pithecia             23               61       0.2411067      0.5581561     0.4770515      1.3657619
## Pongo                23               60       0.2371542      0.4504611     0.5263511      1.2605556
## Propithecus          24               60       0.2173913      0.3704365     0.5004346      1.6368056
## Saimiri              23               62       0.2450593      0.5222637     0.5696146      0.9677419
## Tarsius              23               65       0.2569170      0.6026613     0.5481987      0.9859172
## Tupaia               24               74       0.2681159      0.5534618     0.4627998      0.5858291
```

```
summary(net.complexity)
```

```
##    Bones (N)    Interactions (K) Compactness (D)  Redundancy (C)   Disparity (H)    Modularity (M)  
##  Min.   :21.0   Min.   :60.00    Min.   :0.2174   Min.   :0.3589   Min.   :0.4625   Min.   :0.5486  
##  1st Qu.:23.0   1st Qu.:61.75    1st Qu.:0.2411   1st Qu.:0.4877   1st Qu.:0.4901   1st Qu.:0.9074  
##  Median :23.0   Median :63.50    Median :0.2464   Median :0.5326   Median :0.5059   Median :0.9815  
##  Mean   :23.2   Mean   :64.30    Mean   :0.2502   Mean   :0.5266   Mean   :0.5185   Mean   :1.0600  
##  3rd Qu.:24.0   3rd Qu.:67.25    3rd Qu.:0.2569   3rd Qu.:0.5828   3rd Qu.:0.5389   3rd Qu.:1.2530  
##  Max.   :24.0   Max.   :74.00    Max.   :0.3048   Max.   :0.6339   Max.   :0.6689   Max.   :1.6368
```

##### 4. Estimating the phylogenetic signal of network parameters

We test the assumption of phylogenetic independence (Ho) of network
parameters measuring modularity (M) and complexity (N, K, D, C, H) of
skull networks using a calibrated phylogeny of Primates.

We perform both Abouheif test and Blomberg test.

If p-value < 0.05 we reject Ho (= there is a phylogenetic signal in that trait).

```
  # load phylogeny and combine traits
tree <- read.tree("primatetree.txt")
plot(tree)
```

```
traits.in.tree <- phylo4d(tree, net.complexity)
  # Abouheif's test
abouheif <- abouheif.moran(traits.in.tree, method = "oriAbouheif", nrepet = 1000, alter = "two-sided")
abouheif$names <- parameter.names
print(abouheif)
```

```
## class: krandtest 
## Monte-Carlo tests
## Call: as.krandtest(sim = matrix(res$result, ncol = nvar, byrow = TRUE), 
##     obs = res$obs, alter = alter, names = test.names)
## 
## Number of tests:   6 
## 
## Adjustment method for multiple comparisons:   none 
## Permutation number:   1000 
##               Test        Obs   Std.Obs     Alter      Pvalue
## 1        Bones (N) 0.46756114 3.4855316 two-sided 0.001998002
## 2 Interactions (K) 0.30998522 2.4520333 two-sided 0.017982018
## 3  Compactness (D) 0.03036873 0.2295683 two-sided 0.799200799
## 4   Redundancy (C) 0.24583775 1.7416025 two-sided 0.077922078
## 5    Disparity (H) 0.10497538 0.8630162 two-sided 0.390609391
## 6   Modularity (M) 0.17575027 1.3437183 two-sided 0.158841159
## 
## other elements: adj.method call
```

```
plot(abouheif)
```

```
  # Blomberg's test
blomberg <- matrix(0, ncol = 4, nrow = 6)
rownames(blomberg) <- parameter.names
colnames(blomberg) <- c("K", "Obs variance", "Exp variance", "p-value")
for (i in 1:6){
        signal <- phylosignal(net.complexity[,i], tree, reps = 1000)
        blomberg[i,] <- as.numeric(signal[1:4])
}
print(blomberg)
```

```
##                          K Obs variance Exp variance    p-value
## Bones (N)        0.4786070 2.055676e-02 2.525063e-02 0.37162837
## Interactions (K) 0.6948346 3.858027e-01 7.115512e-01 0.03996004
## Compactness (D)  0.1961156 2.340261e-05 1.757856e-05 0.83116883
## Redundancy (C)   0.3745160 2.150931e-04 2.998424e-04 0.14785215
## Disparity (H)    0.2558823 1.661558e-04 1.273957e-04 0.78221778
## Modularity (M)   0.3406290 3.127194e-03 4.057153e-03 0.17682318
```

##### 5. Testing the Near-decomposability Hypothesis

According to Simon’s hypothesis, modularity facilitates the emergence
of complexity; thus, modularity and complexity should be positively
correlated.

We test the Ho: modularity~complexity r = 0, against the Ha: modularity~complexity r > 0.

If p-value < 0.05, we reject Ho (i.e. there is a near-decomposability effect).

```
  # compute phylogenetic independent contrast
PIC <- vector("list", 6)
names(PIC)<- parameter.names
for (i in 1:6){
        PIC[[i]] <- pic(net.complexity[,i], tree) # phylogenetic independent contrast
}
  # Pearson's test
cor.MN <- cor.test(PIC$Modularity, PIC$Bones, alternative = "g", method = "p")
cor.MK <- cor.test(PIC$Modularity, PIC$Interactions, alternative = "g", method = "p")
cor.MD <- cor.test(PIC$Modularity, PIC$Compactness, alternative = "g", method = "p")
cor.MC <- cor.test(PIC$Modularity, PIC$Redundancy, alternative = "g", method = "p")
cor.MH <- cor.test(PIC$Modularity, PIC$Disparity, alternative = "g", method = "p")
```

```
##                  r      p-value     CI<95% CI>95%
## N vs. M  0.6908061 0.0005288043  0.4122199      1
## K vs. M -0.4416633 0.9708311563 -0.7091683      1
## D vs. M -0.7011651 0.9995881661 -0.8566985      1
## C vs. M -0.4096686 0.9592344907 -0.6891979      1
## H vs. M  0.1490309 0.2712869847 -0.2552907      1
```

Results indicate that modularity and complexity might show an inverse relationship.

We test now the Ho against this alternative hypothesis, Ha: modularity~complexity r < 0.

```
##                  r      p-value CI<95%      CI>95%
## N vs. M  0.6908061 0.9994711957     -1  0.85125958
## K vs. M -0.4416633 0.0291688437     -1 -0.06299832
## D vs. M -0.7011651 0.0004118339     -1 -0.42875914
## C vs. M -0.4096686 0.0407655093     -1 -0.02399488
## H vs. M  0.1490309 0.7287130153     -1  0.50898768
```
